# Bmi1 Is Expressed in Postnatal Myogenic Satellite Cells, Controls Their Maintenance and Plays an Essential Role in Repeated Muscle Regeneration

**DOI:** 10.1371/journal.pone.0027116

**Published:** 2011-11-09

**Authors:** Lesley G. Robson, Valentina Di Foggia, Aleksandar Radunovic, Katy Bird, Xinyu Zhang, Silvia Marino

**Affiliations:** 1 Blizard Institute, Barts and The London School of Medicine and Dentistry, Queen Mary University of London, London, United Kingdom; 2 Neuroscience Clinical Academic Unit, Barts and The London NHS Trust, Whitechapel, London, United Kingdom; Ohio State University, United States of America

## Abstract

Satellite cells are the resident stem cell population of the adult mammalian skeletal muscle and they play a crucial role in its homeostasis and in its regenerative capacity after injury. We show here that the Polycomb group (PcG) gene Bmi1 is expressed in both the Pax7 positive (+)/Myf5 negative (−) stem cell population as well as the Pax7+/Myf5+ committed myogenic progenitor population. Depletion of Pax7+/Myf5− satellite cells with reciprocal increase in Pax7+/Myf5+ as well as MyoD positive (+) cells is seen in Bmi1−/− mice leading to reduced postnatal muscle fiber size and impaired regeneration upon injury. Bmi1−/− satellite cells have a reduced proliferative capacity and fail to re-enter the cell cycle when stimulated by high serum conditions *in vitro*, in keeping with a cell intrinsic defect. Thus, both the *in vivo* and *in vitro* results suggest that Bmi1 plays a crucial role in the maintenance of the stem cell pool in postnatal skeletal muscle and is essential for efficient muscle regeneration after injury especially after repeated muscle injury.

## Introduction

Satellite cells are the main stem cell population of the adult skeletal muscle, they account for between 1–4% of the nuclei associated with adult muscle fibers [1], [2] and they are derived at least in part from Pax7 positive progenitor cells during myogenesis [3], [4]. The satellite cells, located beneath the basal lamina of adult muscle fibers, are normally arrested in G0 of the cell cycle, but can be activated to re-enter the cell cycle in response to physiological stimuli, such as exercise, injury and stress, initiating a well-orchestrated regenerative process, capable of restoring the normal cytoarchitecture of muscle within 2 weeks (reviewed in [5]). The transcription factor Pax7, surface protein CD34, c-Met/scatter factor receptor, M-cadherin and syndecans 3 and 4 are established markers of the quiescent satellite cell population (reviewed in [6]). A “stem” and a “progenitor” satellite populations have been identified [7], the former identified by the expression of Pax7 and no expression of the muscle determining bHLH transcription factor family member Myf5, while the latter express both Pax7 and Myf5 [7]. Upon injury both satellite cell populations become activated and they also switch on the expression of another member of the muscle determining bHLH transcription factor family, MyoD [8]. A proportion of the activated, proliferating satellite cells will down regulate Pax7 expression, become terminally differentiated and begin to express myogenic contractile proteins, fusing with existing fibers or themselves to regenerate damaged areas [9]. However, as the number of satellite cells remains relatively constant throughout adult life, a proportion of the activated satellite cells down regulate MyoD and return to a quiescent state [10]. Therefore, satellite cells can be regarded as a myogenic stem cell population, able to self-renew as well as to give rise to differentiated muscle fibers.

Despite recent advances in identifying new molecular markers of satellite cells, much remains to be elucidated on the signaling pathways that control their self-renewal and activation in response to physiological and pathological conditions throughout the life span of an organism. While survival and expansion of muscle progenitors during embryonic development depends on Pax7 [11], the role of Pax7 in the postnatal satellite cell population has been recently questioned. Even though Pax7 expression is retained in satellite cells, its conditional ablation after 2/3 weeks of age had no effect on the ability of muscle to grow and regenerate after injury in a mouse model [12]. Other factors could therefore be responsible for the stem cell behavior of satellite cells.

Polycomb group (PcG) genes are epigenetic chromatin modifiers involved in heritable gene repression and maintenance of stem cell self-renewal and proliferation [13], [14]. Expression of Ezh2 is developmentally regulated in skeletal muscle and prevents myogenic gene transcription and differentiation, keeping myogenic cells in a committed yet undifferentiated state [15], [16]. Conversely, Epc1 increases the expression of myogenic determination factors and is involved in initiation of muscle differentiation [17]. However, the role of other PcG genes in self-renewal, activation and differentiation of satellite cells has not been studied in detail.

Bmi1 has been shown to play a crucial role in regulating cell cycle entry as well as in maintaining self-renewal capacity of various adult stem cells [13], [14], [18]. These actions are, at least in part, mediated through transcriptional repression of the *INK4A-ARF* locus, encoding cell cycle inhibitors *p16^ink4a^* and *p19^arf^* [13], and *p21^waf1/cip1^* [19]. Interestingly it has been reported that loss of p16^ink4a^ induces spontaneous immortalization of myogenic cells [20] and satellite cells can be conditionally immortalized by constitutive expression of Bmi1 and telomerase [21]. It is therefore possible that Bmi1 plays a crucial role in maintaining the proliferative potential of satellite cells, possibly through its actions on p16^ink4a^ and p19^arf^. In agreement with this hypothesis, inhibition of the *INK4a-ARF* locus by Bmi1 has been shown to be dependent on the continued association with Ezh2. Down-regulation of Ezh2 leads to displacement of Bmi1 and activation of *INK4a-ARF* transcription resulting in senescence [22]. Moreover, post transcriptional inhibition of Ezh2 through miRNA-26a is crucial in inducing myogenic differentiation *in vitro* [23] and conditional deletion of Ezh2 in Pax7+ cells and their progeny leads to reduced muscle mass and impaired regeneration [24].

In this study, we show that Bmi1 is expressed in human and murine satellite cells. While Bmi1 was dispensable for embryonic myogenic development, severe depletion of the postnatal stem cell compartment was observed, which was due to an impaired repopulation of the stem cell pool after activation. Functionally, impaired muscle regeneration after injury was noted, a phenomenon which was particularly prominent after repeated injury.

## Materials and Methods

### Ethics statement

All procedures on animals had Home Office approval (Animals Scientific Procedures Act 1986, PPL 70/6452). The use of human muscle samples in this research was in agreement with the UK Human Tissue Act 2004 (http://www.hta.gov.uk/legislationpoliciesandcodesofpractice/legislation/humantissueact.cfm) and its use for this specific research had ethical approval (East London and The City REC Alpha, ReDA Reference: 006244).

### Satellite cell culture

Single fibers from adult Bmi1+/+ (wild type) and Bmi1−/− soleus muscles were isolated and cultured to obtain pure cultures of satellite cells. Briefly, the soleus was dissected from P60 mice being careful not to stretch or stress the muscles. Tendon and other connective tissue was carefully removed and the muscles incubated in 0.2% collagenase type 1 (Worthington) at 37oC for 2 hr. Fibers were liberated by gentle trituration in DME medium. Single fibers were placed in a 24 well plate (BD Falcon) coated with Matrigel (1 mg/ml in DME, BD Biosciences). Single fibers were allowed to adhere for 5 min at 37oC before 500 µl of plating medium was added (DME supplemented with 10% horse serum (PAA) 0.5% chick embryo extract (GIBCO) with antibiotics) and incubated at 37oC 5% CO2. After 3 days the satellite cells had started to leave the fibers and the media was changed to proliferation media (DME supplemented with 20% FCS (PAA) 2% chick embryo extract (GIBCO) plus antibiotics). Satellite cells were plated at 2000 cells per well in an 8 well chambered slides (BD Falcon) and plated in differentiation media (DME supplemented with 2% horse serum and antibiotics), and cultured for 2 or 5days then switched back to the proliferation media for 2 days.

### BrdU incorporation

Myoblast cultures from P7 and P60, wild type, and Bmi1−/−, in proliferation, differentiation media for 2 or 5 days or after returning to the proliferation media after 5 days in differentiation media, were pulsed for 2 hrs with 100 µM BrdU (final concentration), and stained using anti-BrdU and Pax7.

### Muscle injury and histological analysis

Bmi1−/− and wild type littermates between 8–12 weeks old were used. Mice were anesthetized using 2% isofluorane inhalation. A 5 mm incision was made overlying the TA muscle and a 2 mm metal probe pre-cooled in dry ice was applied to the surface of the exposed muscle for 10 sec to induce the muscle damage. The incision was closed by application of VETBOND™ Tissue Adhesive. Mice were sacrificed 10 or 21 days after the injury. Additionally a group of mice underwent a second injury on the same area of the TA 21 days after the first injury and then sacrificed a further 10 days later. The lower limb muscles were removed, and snap frozen in isopentane cooled by liquid nitrogen. The muscles were serial sectioned at 8 µm for H&E. The proportion of fibers with central nuclei (regenerating fibers) was counted in the injured area and the cross-sectional areas (CSA) of the fibers in the non- and injured areas were measured using the MetaMorph software (Life Sciences Research Imaging Systems).

### Immunohistochemistry

All myoblast cultures and muscle sections were fixed with 4% PFA for 4 min at 4oC. Standard H&E procedures were used for sections. For immuno-labelling primary antibodies were applied overnight at room temperature, appropriate fluorescent secondary antibodies were used and then the myogenic cultures or sections were mounted using vectorshied+DAPI. All primary and secondary antibodies used are presented in the supplementary section.

### Forelimb grip strength measurements

Wild type and Bmi1−/− mice were subjected to forelimb grip strength tests using a horizontal grip strength meter (Linton). Mice were lowered by the tail towards the metal pull bar on the apparatus. Once the mice had grasped the bar with their forelimbs they were pulled backwards in the horizontal plane. The procedure was repeated consecutively five times with 30 seconds of rest between the pulls. The body weight of the animals was taken so that the relative force for the body weight could be calculated.

### Microscopy and quantification

Fluorescent and bright field microscope and image capture were performed using a Leica epifluorescent microscope or Zeiss Meta 510LSM confocal. Confocal images were opened using the Zeiss LSM software and the individual channels taken into Adobe Photoshop CS3. Images were optimized for brightness and contrast and assembled into figures using Adobe photoshop CS3 (Adobe Systems Incorporated).

In myoblast cultures, counts were made of all cells assessed by DAPI labeling and the proportion of positive Pax7, MyoD and MyHC positive cells counted. Data from multiple myoblast cultures or myofiber cultures were pooled to give a population mean +/− SEM. Significance was tested by Student's t-Test.

## Results

### Bmi1 is expressed in satellite cells in the postnatal murine and human skeletal muscle

Satellite cells are specified prior to birth and are characterized by the expression of Pax7 [25]. In early postnatal muscle they account for around 30% of the sublaminar nuclei, but as muscles grow the population of satellite cells falls to around 1–4% of adult muscle nuclei, this percentage then remains fairly constant throughout life [26].

**Figure 1:**
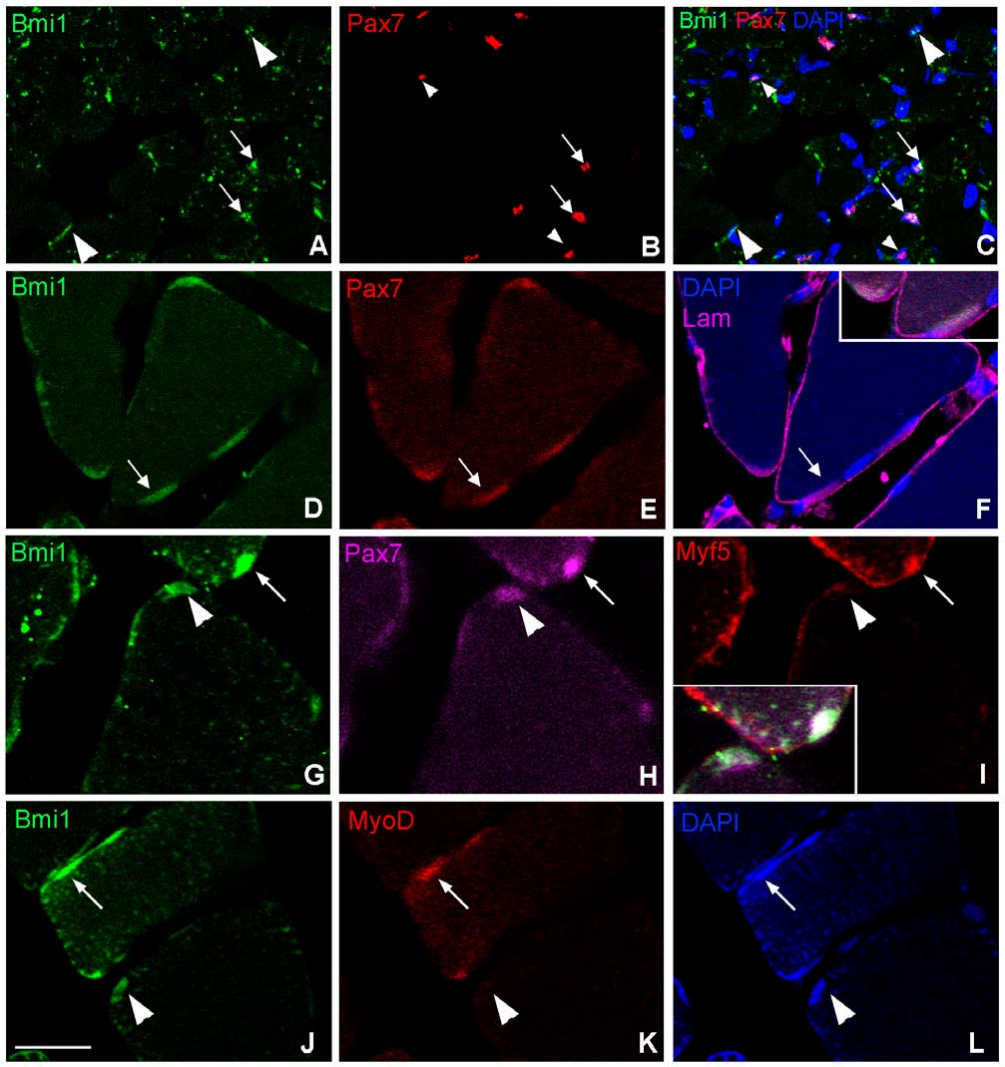
Characterization of Bmi1 expression in the murine skeletal muscle. In the skeletal muscle at P7, the majority of Bmi1+ cells (A) also co-express Pax7 (B and C arrows) while only occasionally there are Bmi1+/Pax7− cells (A and C arrowheads). As expected for transcription factors both the Pax7 and Bmi1 labeling overlies the nuclei, visualized with DAPI (C). At P60 all Pax7+ cells also express Bmi1 (arrow D–F and inset in F of arrowed cell). The Pax7+/Bmi1+ cells are identified as satellite cells by their location underneath the basal lamina visualized by laminin labeling (F); myonuclei are negative for Pax7 and Bmi1 but can be seen as DAPI positive (F). Triple immunostaining shows that the committed progenitor satellite cell population which are Pax7 positive and Myf5 positive also express Bmi1 (G–I arrow and inset in I), while the “stem” cell satellite population only expresses Bmi1 and Pax7 and are negative for Myf5 (G–I arrowhead and inset in I). Therefore, Bmi1 marks both the stem and progenitor populations of satellite cells in the adult skeletal muscle. A proportion of Bmi1 positive cells also express the bHLH myogenic determination factor, MyoD (J–L arrow). These are either activated satellite cells that contribute to muscle maintenance in the adult or early-differentiated myonuclei. However, the majority of Bmi1 positive cells are MyoD negative, suggesting that Bmi1 marks mainly the satellite cell population (arrowhead-identified cell J–L). Scale bar 25 µm.

Here, we set out to characterize the expression of Bmi1 in the murine soleus muscle (SOL) at two time points, early postnatally at day 7 (P7) and in the adult at day 60 (P60). We show that Bmi1 is expressed in a substantial proportion of nuclei at both time points (Fig. 1A and D). Dual immunofluorescence for Pax7 and Bmi1 revealed that the majority of Bmi1+ cells were also Pax7+ (Fig. 1A–H) and confirmed that the proportion of Pax7+/Bmi1+ cells was larger at P7 compared with P60. At P7 and P60 Bmi1+/Pax7+ cells were clearly located beneath the basal lamina, as assessed by triple immunolabeling with laminin, Pax7 and Bmi1 (Fig. 1F and inset of cells shown by arrow and data not shown). At P7 we could identify a small proportion of Bmi1−/Pax7+ cells (Fig. 1A–C small arrowheads) and a subpopulation of Bmi1+/Pax7− cells could be identified both at P7 and at P60 (Fig. 1A–C large arrowheads). The latter were located in the interstitial spaces between the muscle fibers or under the basal lamina of the muscle fiber at P60, in keeping with interstitial cells and differentiated myonuclei respectively (Fig. 1A–C large arrowheads).

Satellite cells are a heterogeneous mixture of a small population of stem cells and a larger number of committed progenitor cells. While all satellite cells express Pax7, co-expression of Myf5 characterizes the quiescent but committed myogenic progenitor population. To assess whether Bmi1 expression was confined to the Pax7+/Myf5− subpopulation or whether it was expressed also in Pax7+/Myf5+ myogenic progenitors, triple labeling with Bmi1, Pax7 and Myf5 was carried out. We show that Bmi1 co-localizes not only with Pax7+/Myf5− stem cells (Fig. 1G–I arrowhead and inset in I) but also with Pax7+/Myf5+ quiescent committed progenitors (Fig. 1 G–I arrow and inset in I). We found that in P60 muscles 15% of the cells positive for Bmi1, were also Pax7+/Myf5− stem cells while 85% of the Bmi1+ cells expressed Pax7 and Myf5. These data are in agreement with the work of Kuang et al. [7] who identified 10% of satellite cells as Pax7+/Myf5−.

To assess whether Bmi1 was also expressed in activated satellite cells, double labeling for Bmi1 and MyoD, a marker of activated satellite cells, was carried out. Occasional cells co-expressing Bmi1 and MyoD were identified, which could be either activated satellite cells or newly differentiated myonuclei (Fig. 1J–L arrows). However, the majority of Bmi1 positive cells were negative for MyoD, in keeping with these being quiescent satellite cells (Fig. 1 J–L arrowheads).

**Figure 2:**
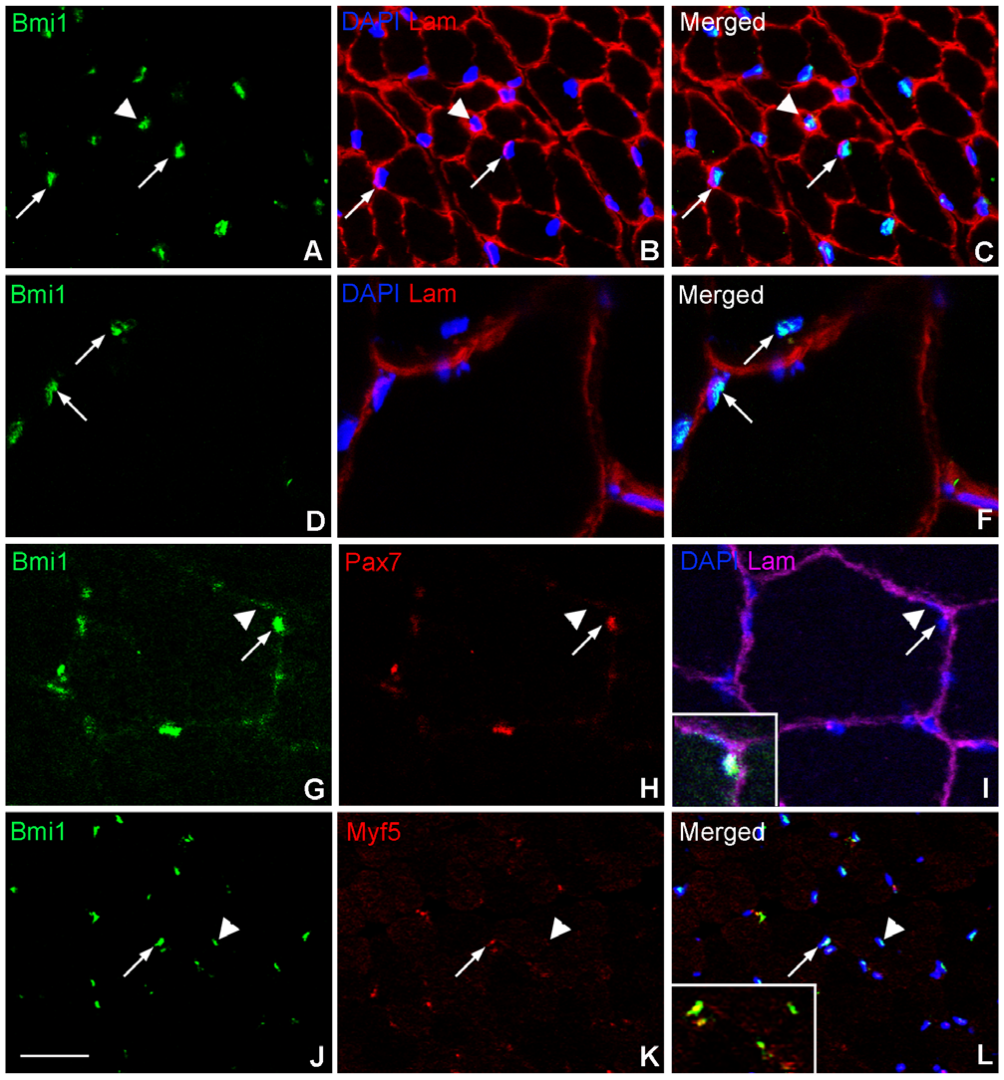
Characterization of Bmi1 expression in human skeletal muscle. Bmi1 is expressed in several sub-laminar nuclei in the skeletal muscle of a 4-year-old child (A–C arrows); the basal lamina is identified by laminin (Lam) labeling. There are also Bmi1+ cells located in the interstitial space between muscle fibers (A–C arrowhead). In the adult skeletal muscle, Bmi1+ cells are also identified in a sub-laminar position (D–F arrows). Bmi1 expression co-localizes with Pax7 expression (G–H arrows and I inset). Weak Bmi1 expression is also seen in the myonuclei of the muscle fibers identified by their sub-laminar position and the absence of Pax7 positivity (G–I arrowhead). Also in human skeletal muscle, a proportion of Bmi1 positive cells co-express Myf5 (J–L and inset arrow), in keeping with committed progenitor pool of satellite cells, while Bmi1+/Myf5− cells are also seen (J–L and inset arrowhead) in keeping with “stem” cell satellite cell sub-population. Scale bar 25 µm.

Finally, we analyzed the expression of Bmi1 in normal human skeletal muscle obtained as part of routine diagnostic procedures for neuromuscular disorders and classified as normal upon neuropathological analysis. Muscle biopsies from a 4-year-old and from a 37- and a 44-year-old were used. In keeping with the results obtained from the murine skeletal muscle, frequent Bmi1 positive cells located underneath the basal lamina of muscle fibers were identified in human muscle at all ages (Fig. 2 A–I arrows) with more Bmi1 positive cells identified in the biopsy from the 4-year-old compared to the two older samples (Fig. 2 A–F).

Double immunolabeling for Bmi1 and Pax7 (Fig. 2 G–I and inset in I) and for Bmi1 and Myf5 confirmed that Bmi1 is strongly expressed in both the quiescent stem cell satellite cell population (Fig. 2 J–L arrowhead and inset in L) and committed progenitor satellite cells in human muscle (Fig. 2 J–L arrow and inset in L). Weakly Bmi1+/Pax7− cells were identified that are likely to be myonuclei.

In summary, these results demonstrate that Bmi1 is robustly expressed in both quiescent and activated satellite cells in both murine and human muscle. Its expression is then down regulated as these cells differentiate into myonuclei where it is only weakly expressed.

### Bmi1 deficiency results in depletion of the satellite cell pool and smaller fiber size *in vivo*

Bmi1−/− mice are born in a Mendelian fashion and are undistinguishable from non-transgenic littermates during embryonic and fetal development and at birth. Postnatally they show growth retardation as well as neurological and haematological abnormalities, as a result of impaired self-renewal and maintenance of postnatal stem cells and impaired proliferation of some committed progenitor cells [27], [28], [29].

We set out to analyze whether myogenic satellite cells are dependent on Bmi1 for their self-renewal and maintenance. We examined skeletal muscles of Bmi1−/− and control littermates at embryonic day 16 (E16) -during the period of fetal myogenesis- at P7 -prior to the onset of the neurological symptoms where no secondary muscle pathology is expected- and at P60 -when the mouse is ataxic due to the central nervous system phenotype [29], [30].

To assess whether loss of Bmi1 affected embryonic myogenesis we counted the number of muscle fibers and of Pax7+ cells in the axial, diaphragm and limb muscles. No significant difference was found in the total number of muscle fibers, in the percentage of Pax7+ cells and in the fiber type patterning in Bmi1−/− and control muscles (Fig. S1 and data not shown). These findings suggest that Bmi1 is not required for the early events in the commitment of myogenic precursors to the myogenic lineage and in their differentiation and muscle patterning.

**Figure 3:**
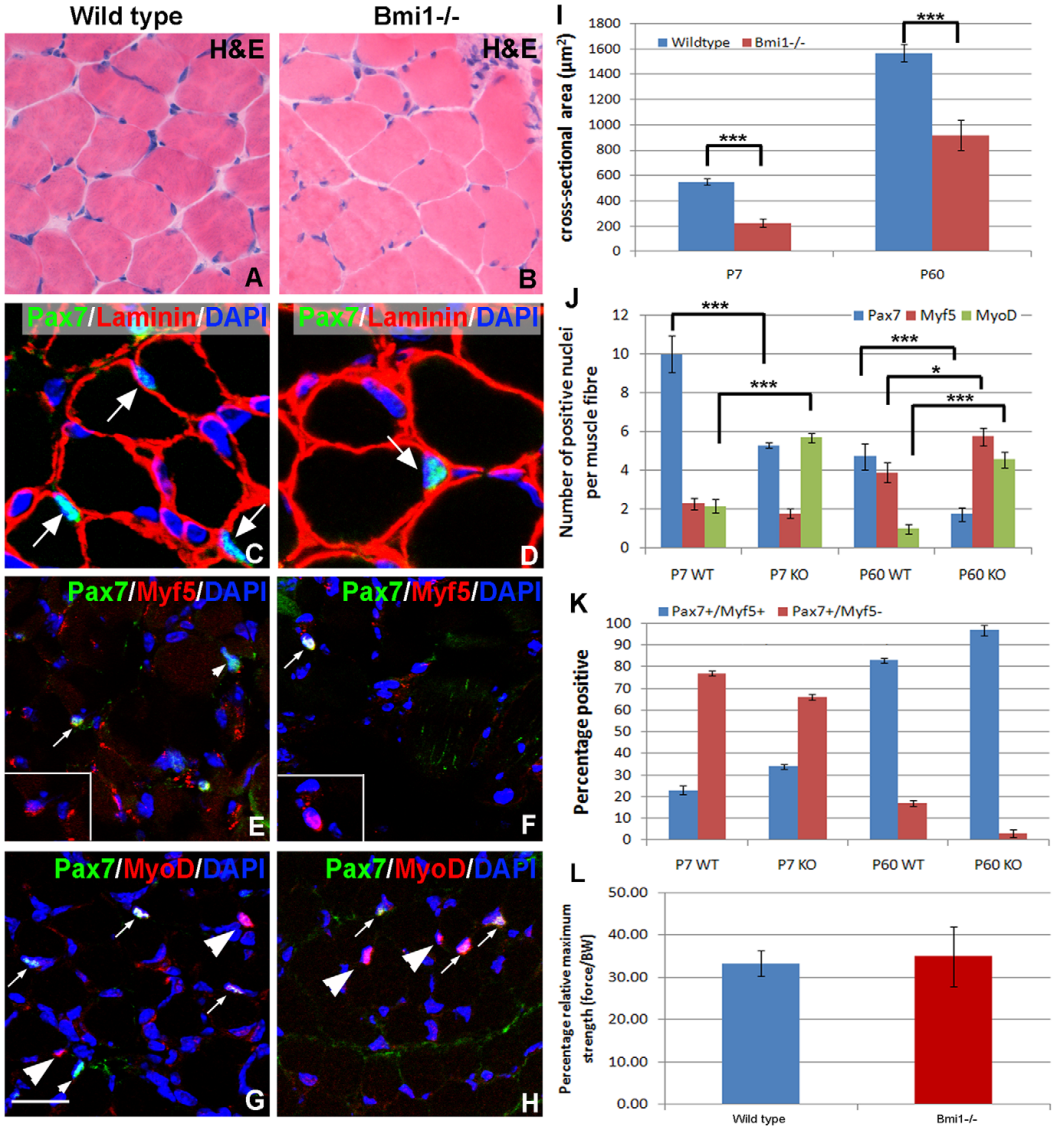
Reduced number of Pax7+ satellite cells in Bmi1−/−skeletal muscle at different postnatal time points. In P60 Bmi1−/− mice the overall cell fiber patterning and the number of the muscle fibers is not significantly different from the wild type. The H&E staining does not show increase in centrally located nuclei or other signs of degeneration and regeneration of the muscle fibers. However, increased variation in the cross-sectional areas of the muscle fibers is seen in the Bmi1−/− muscles (A–B and I). Staining for Pax7 reveals reduced numbers of Pax7 positive cells at P60 (C–D arrows and quantification J) and at P7 (E–H arrows and quantification J). There is a small increase in the proportion of Myf5 positive cells in the Bmi1−/− muscles, which is mainly seen at P60 rather than in the P7 Bmi1−/− muscles (E–F and J–K). While there is a significant increase in the number of Pax7+/Myf5+ in Bmi1−/− mice, the number of Pax7+/Myf5− cells is reduced (K) in comparison with wild type littermates. There is also a significant reciprocal increase in the number of MyoD positive cells in Bmi1−/− muscles at both P7 and P60 (G–H arrowheads and quantification J). Generally the muscle fibers have smaller cross sectional areas in Bmi1−/− muscles at both P7 and P60 (P7 n = 6, P60 n = 5 A–B and quantification I). Lam represents laminin labeling. Scale bar = 50 µM. * <P0.05%, ** <P0.01% and *** <P0.001. Analysis of the relative grip strength (L) shows that there is no significant difference between the Bmi1−/− and wild type animals suggesting that although smaller the muscles fibers are functionally normal. Values are mean ± SEM (* P<0.05).

At P7, the number of Pax7+ satellite cells was 11.2 of the total nuclei per muscle fiber, while in Bmi1−/− muscles it was 5.5 of the total nuclei per muscle fiber (Fig. 3C–D and J). At P60 the decline in the total number of Pax7+ satellite cells had continued in the Bmi1−/− muscles, with 1.8 of the total fiber nuclei Pax7+ compared to 4.7 in wild type muscles (Fig. 3J). This reduction in the number of Pax7+ cells was statistically significant at both P7 and at P60 (P = <0.001, Fig. 3J) and it is in keeping with a severe postnatal loss of satellite cells in the Bmi1−/− muscle.

Next, we set out to assess whether Bmi1 deficiency would differentially affect the Pax7+/Myf5− stem cell population and the Pax7+/Myf5+ myogenic cell population. We show that at P7 the percentage of Pax7+/Myf5− satellite cells is reduced in Bmi1−/− muscle (76% wild type compared to 65% Bmi1−/− Fig. 3E–F and K) while the percentage of Pax7+/Myf5+ committed myogenic progenitors is slightly but significantly increased (23% wild type compared to 35% Bmi1−/− Fig. 3K). At P60 the percentage of Pax7+/Myf5− satellite cells has declined with a reciprocal increase in the proportion of satellite cells that have been activated and which have returned to the quiescent pool (Pax7+/Myf5+) in both Bmi1−/− and controls. At this time point the decline in the Pax7+/Myf5− stem cell population in the Bmi1−/− muscle is even more dramatic (3% in Bmi1−/− as compared to 15% in the wild type muscle, Fig. 3K). These data were statistically significant at both time points (P7 P = <0.01 and P60 P = <0.001). These data raise the possibility that Bmi1 deficiency impairs the maintenance of the satellite cell pool.

Normally the myogenic determination factor, MyoD is quickly down regulated soon after birth as activated myogenic cells differentiate and fuse with existing muscle fibers, a phenomenon which is responsible for the rapid growth of muscles postnatally. At P7, we observed a considerable higher number of MyoD+ nuclei in Bmi1−/− muscles compared to control muscles (5.8 positive nuclei per muscle fiber compared to 2.1 respectively (Fig. 3G–H and J)). This increased proportion of MyoD+ cells is also observed at P60, (4.6 of the total number of muscle fiber nuclei in the Bmi1−/− versus 1 in the wild type muscle (Fig. 3G–H and J)). These data were statistically significant at both time points (P = <0.001). We conclude that although there is a reduction in the number of Pax7+ cells in the Bmi1−/− mice there is a reciprocal increase in the number of Myf5+ committed as well as MyoD+ committed and activated satellite cells, in keeping with failure to re-populate the satellite stem cell pool.

To confirm that the reduction in the number of satellite cells was affecting muscle growth, morphological and histochemical analysis was carried out on P7 and P60 SOL muscles from Bmi1−/− and wild type littermates. Morphological analysis on H&E stained sections revealed that the size of the muscle fibers was more variable in Bmi1−/− mice and that the number of smaller fibers was increased in Bmi1−/− muscles compared to control littermates at both P7 and P60 (Fig. 3A–B and I). At P60 labeling for laminin showed that the sarcolemma appeared thicker and rougher in the Bmi1−/− SOL muscle compared to the wild type, raising the possibility that there is impaired regeneration in the Bmi1−/− muscle (Fig. 3C–D). Mild increase of the endomysial connective tissue was also noted with a Gomori-Trichrome stain (data not shown). No difference in the number of centrally located nuclei was noted in P60 Bmi1−/− muscles compared to wild type (Fig. 3A–B), suggesting that there is no myopathic degenerative changes in the Bmi1−/− muscles. Importantly, there was no evidence of denervation and reinnervation, which would be a sign of secondary muscle damage to a primary neural pathology. In addition, we were unable to detect any differences in the relative grip strength between the wild type and Bmi1−/− mice once the overall smaller size of the Bmi1−/− mice was taken into consideration (Fig. 3L). These data suggest that the Bmi1−/− mice do not have a primary or a secondary muscle defect and that the muscles that form are structurally and functionally normal. However, loss of Bmi1 results in a reduced number of satellite cells raising the possibility that their function may also be compromised, therefore explaining the variation in the cross-sectional area of the muscle fibers observed. Finally, the reduced and impaired satellite cells in the Bmi1−/− mice may be responsible for the reduced postnatal growth of the muscle mass and therefore contribute to the reduced body size of these animals.

Finally, deletion of Bmi1 did not affect the expression of other PcG genes known to play a role in myogenesis. Indeed qRT-PCR analysis did not reveal significant differences in the expression of Ring1B, Mel18, Ezh2 and Suz12 (Fig. S2).

### Muscle regenerative capacity is reduced in Bmi1-deficient mice

To test functionally the impact of a depleted stem cell pool on the regenerative capacity of the adult skeletal muscle, two experimental model systems were chosen, the freeze injury model [31] and the mdx dystrophinopathy model [32]. These two experimental settings were chosen as an example of an acute traumatic muscle injury and a chronic cell type specific muscle injury, where the lack of dystrophin causes continuous activation signal to satellite cells, with repeated degeneration-regeneration cycles and exhaustion of both satellite cells pool and skeletal muscle, respectively.

Acute freeze injury was induced in the tibialis anterior (TA) muscle of adult (P30) Bmi1−/− mice and wild type littermates and the repair to injury was assessed 10 days and 21 days later. These time points were chosen as they represent the mid-point and the end-point of the regeneration respectively.

**Figure 4:**
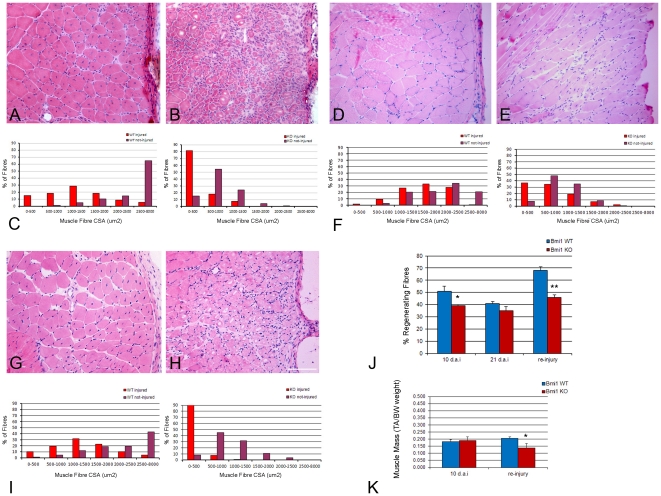
Regeneration after acute freeze injury is delayed in the Bmi1−/− muscle. H&E stained sections of regenerating TA muscle 10 days after a freeze injury in wild type (A) and Bmi1−/− (B). The regenerating area contains muscle fibers with central nuclei in both wild type and Bmi1−/− muscles but the size of the regenerating fibers in the wild type are significantly larger than in the Bmi1−/− (A–C). Measurement of cross-sectional area (CSA; µm2) of the fibers in uninjured (control) and injured areas reveals a more prominent shift towards smaller fibers in Bmi1−/− in comparison with wild type after muscle injury, suggesting a slower/impaired regenerative process in the Bmi1−/− muscle. H&E stained sections of regenerating TA muscle 21 days after a freeze injury in wild type (D) and Bmi1−/− (E), also shows that there are significantly more centrally located nuclei in Bmi1−/− muscle compared to wild type. There are also areas of thickened endo- and peri-mysium in the Bmi1−/− muscle identified by the greater white areas between the muscle fibers (D–E). Measurement of cross-sectional area at this time point reveals that wild type muscles have regained the profile of fiber sizes seen in uninjured areas while there are still significantly smaller fibers in Bmi1−/− muscles even though this increase is less prominent in the latter (F). H&E stained sections of regenerating TA muscle 10 days after a second round of freeze injury on the same area of TA injured in the first round in wild type (G) and Bmi1−/− (H) mice. While regeneration in the wild type animals is similar to that after the first injury and may even be slightly improved (G), it is significantly delayed in Bmi1−/− mice, as assessed by the persistence of vacuolated fibers and the infiltration of inflammatory cells (H). Measurement of cross-sectional areas reveals that there is a more prominent shift towards smaller fibers (91% in the second injury against 81% in the first injury) in Bmi1−/− in comparison with the wild type where the phenotype does not show any worsening as compared to the one in the first injury (compare C with I). (J) Quantification of the percentage of centrally nucleated fibers in injured areas at 10, 21 days after injury and 10 days after re-injury shows that there are significantly fewer regenerating fibers at 10 days and 10 days after the second injury in the Bmi1−/− TA muscle compared to the wild type. There is no difference in the number of centrally located nuclei at 21 days after the initial injury. Values are mean percentages ± SEM (* P<0.05 and ** P<0.01). (K) Contra-lateral TA muscle mass 10 days after injury and 10 days after re-injury indicates that there is a significant reduction even in the contra-lateral TA muscle mass 10 days after the second round of injury. Scale bar 25 µm.

10 days after injury, H&E staining of the injured TA muscles revealed significantly fewer regenerating fibers, which could be clearly identified by their centrally located nuclei, in Bmi1−/− muscles (P<0.05, Fig. 4A–B and quantification J). 21 days after injury though, the number of centrally nucleated fibers was similar in Bmi1−/− and control muscles (Fig. 4 D–E and quantification J). Assessment of the cross-sectional areas (CSA) of the muscle fibers in both injured and uninjured regions of the TA muscle revealed an even distribution in the size of the muscle fibers in both regions (Fig. 4C–F). However in the Bmi1−/− TA muscle the majority of the regenerating fibers were very small, importantly even smaller than uninjured Bmi1−/− muscle fibers (Fig. 4C–F), an observation which was significant at both time points analyzed. These results suggest that there is a delay in the regeneration of the Bmi1−/− muscles after an acute traumatic injury.

**Figure 5:**
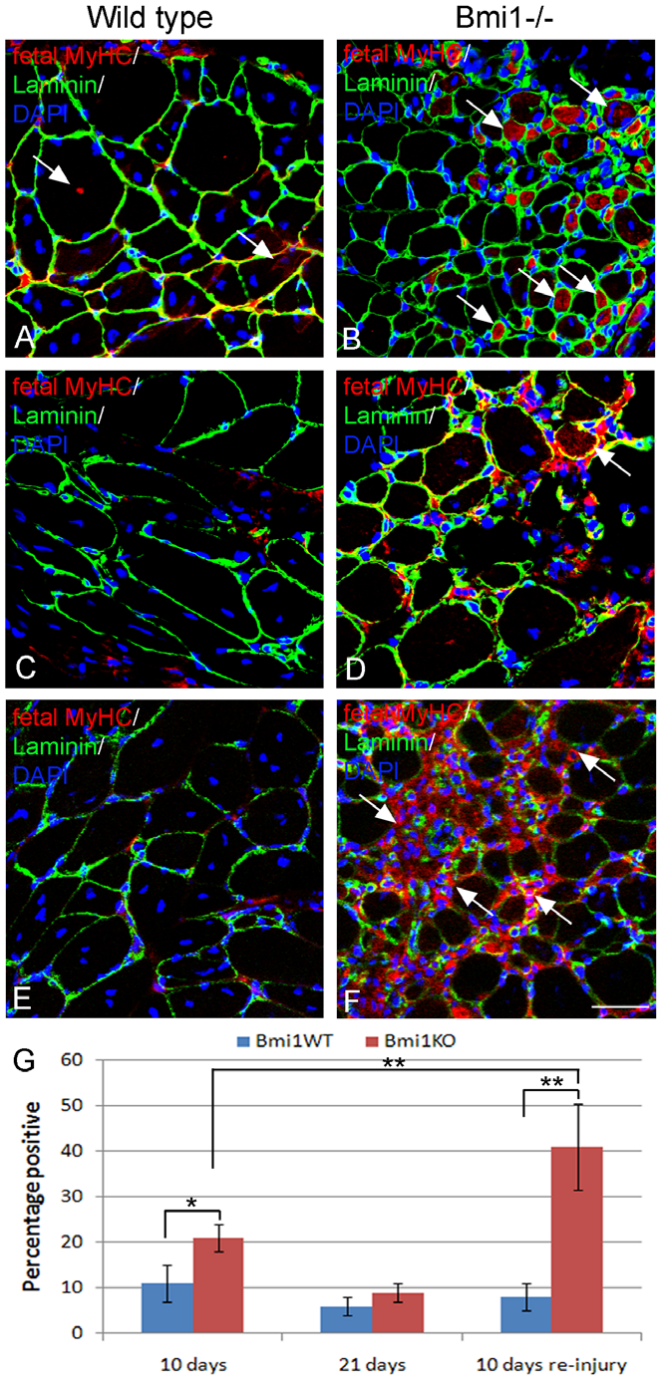
The expression of fetal MyHC after acute freeze injury confirms delayed regeneration in the Bmi1−/− muscle. Fetal MyHC immunolabelling of wild type and Bmi1−/− TA 10 days after an acute freeze injury shows that there are very few and weakly positive fibers in the wild type muscle (red cytoplasmic labeling in A (arrows), laminin green demarcates the basal lamina of the fibers, DAPI (blue) labels the nuclei). In comparison, there are still clearly visible fetal MyHC+fibers in the TA of the Bmi1−/− animals (B, arrows). By 21 days after the initial injury there are very few positive muscle fibers in both wild type and Bmi1−/− muscles (C–D). When a second round of injuries is performed, the Bmi1−/− muscles show a significant increase in the proportion of fetal MyHC+fibers 10 days after the second injury compared to the wild type that has a similar proportion as seen in after the initial round of injury (E–F). (G) Quantification of the proportion of fetal MyHC+fibers in the wild type and Bmi1−/− muscles shows that there is a slower regeneration even after the initial injury but that this is significantly worse after the second round of injury. (* P<0.05, ** P<0.01). Scale bar 25 µm.

To provide further support to this conclusion the number of fibers expressing the fetal isoform of the myosin heavy chain (MyHC) was assessed after freeze injury in Bmi1−/− mice and controls. The regenerating fibers undergo the same sequence of MyHC expression as during development. In fact, regenerating fibres express fetal MyHC initially and then switch to adult isoforms while they mature. In the wild type TA, 10 days after the freeze injury, very few fetal MyHC+fibers were found, while in the Bmi1−/− TA significantly more fetal MyHC+fibers were identified (P<0.05, Fig. 5A–B and quantification G). The fetal MyHC+fibers were also more strongly positive in the Bmi1−/− muscles compared to the weak and patchy labeling in the wild type muscles (Fig. 5A–B). By 21 days after the initial injury, there was no significant difference between the wild type and Bmi1−/− TA muscles (Fig. 5C–D and quantification G), although few fetal positive muscle fibers were still observed in the Bmi1−/− muscles (Fig. 5D arrow).

To test the hypothesis that repopulation of the stem cell pool is the main satellite cell function affected by Bmi1 deficiency, we set out to perform a second round of freeze injury in the same area of the TA 21 days after the first injury. We show a dramatic shift of CSA toward smaller fibers in the Bmi1−/− muscle as compared to re-injured controls (Fig. 4G–H and I). Moreover, significant reduction in the muscle mass was seen in the contralateral TA muscle of Bmi1−/− mice after re-injury as compared to its weight after a single injury (P<0.05; Fig. 4K). The analysis of the fetal MyHC expression 10 days after a further round of injury shows that there were significantly more fetal MyHC+fibers in the Bmi1−/− muscles compared to the wild type (Fig. 5E–F and quantification G). Interestingly, far more fetal MyHC+fibers were noted in the Bmi1−/− 10 days after the re-injury as compared to 10 days after the initial injury (Fig. 5G), whereas in the wild type the proportion of fetal MyHC+fibers was similar (Fig. 5G). These data suggest that the regeneration potential of the Bmi1−/− muscles is impaired particularly in the context of a repeated injury to the muscle.

**Figure 6:**
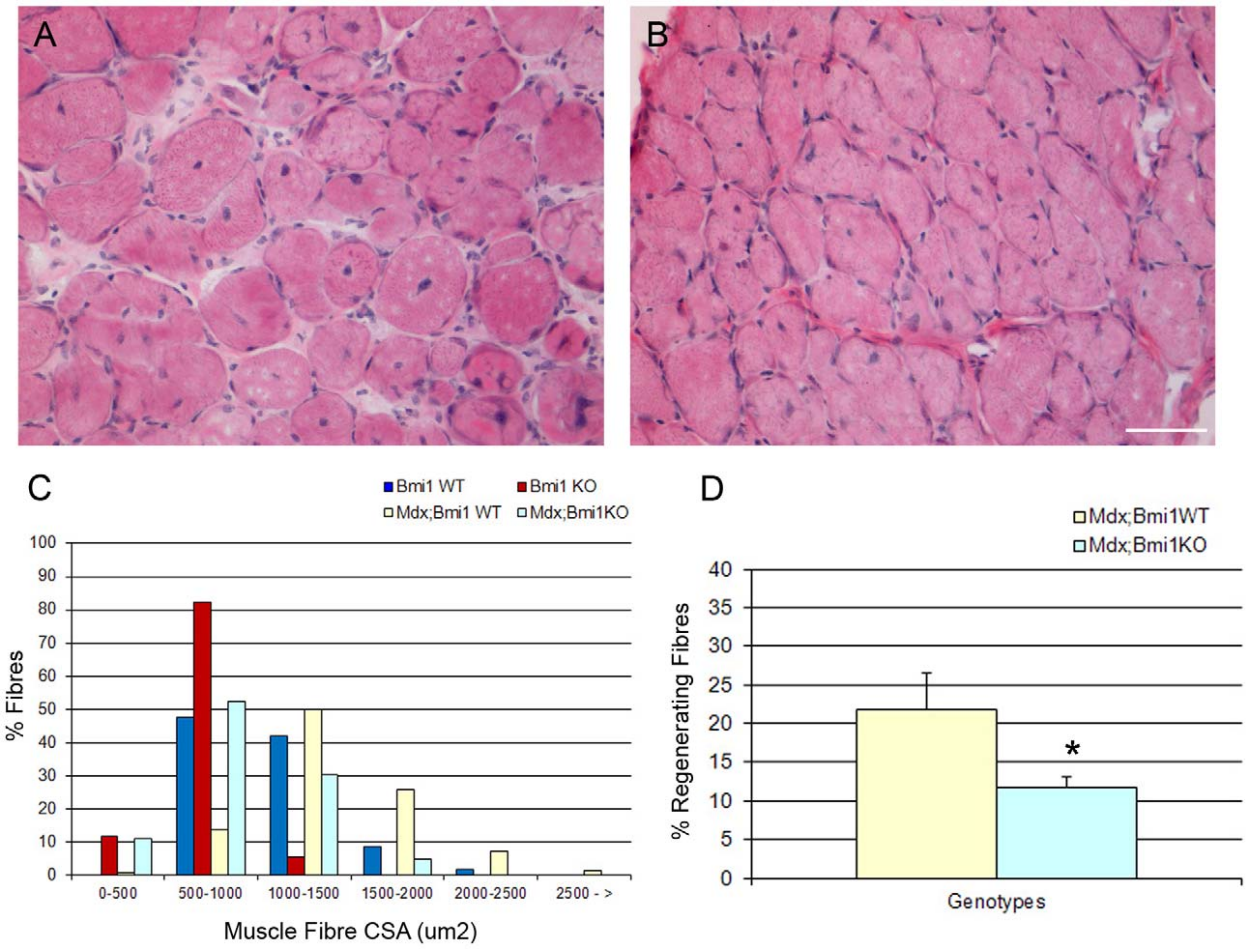
Regeneration is compromised in Mdx;Bmi1−/− muscle. H&E stained sections of diaphragm of P30 Mdx (A) and Mdx;Bmi1−/− mice (B) show that in the Mdx;Bmi1−/− there are fewer centrally nucleated fibers compared to the Mdx diaphragm, suggesting an impaired regenerative potential in the Mdx:Bmi1−/− muscle (D), (* P<0.05). (C) Measurement of cross-sectional area (µm2) of the fibers in wild type, Bmi1−/−, Mdx and Mdx;Bmi1−/− mice show that the loss of Bmi1 causes a shift to smaller fibers in the Mdx mouse (C). Scale bar 25 µm.

The mdx mouse is a mouse model of dystrophynopathy which carries a premature stop codon in exon 23 of the dystrophin gene and has no detectable levels of dystrophin protein in muscle tissue, except in rare revertant fibers [33], [34]. We have crossed this mouse with Bmi1+/− mice to obtain Mdx;Bmi1−/− mice and the number of regenerating fibers as well as CSA of the muscle fibers was assessed in the diaphragm at P30 (Fig. 6). This particular muscle was chosen as it has been shown to be particularly affected by chronic and repeated injury due to its essential role in respiration [35], [36], [37]. We show reduced number of regenerating fibers and a shift of the CSA toward smaller fibers in a very similar fashion to what observed in the freeze injury model (Fig. 6C in comparison with Fig. 4).

Taken together these findings indicate that the regenerative process is not as efficient in Bmi1-deficient muscles and that it is particularly compromised in the context of a repeated injury, which relies on a replenished stem cell pool.

### Bmi1−/− satellite cells proliferative capacity is reduced *in vitro*

**Figure 7:**
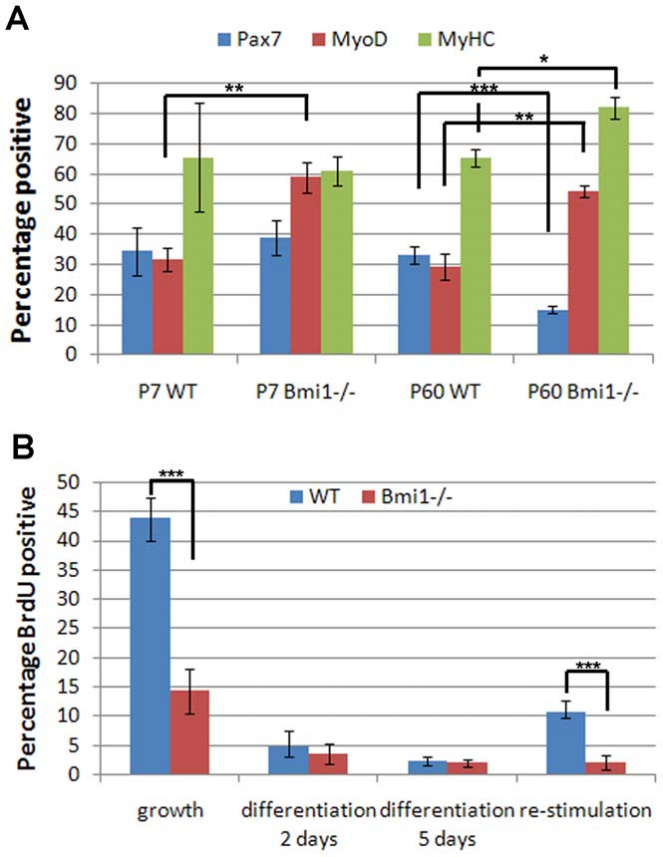
Bmi1−/− satellite cells proliferative capacity is reduced *in vitro*. There are fewer Pax7+ cells in the Bmi1−/− satellite cell cultures and an increase in the proportion of MyoD+ and MyHC+ cells both at P7 and P60 (n = 4, A). Labeling with BrdU to identify the proportion of proliferating satellite cells indicates that Bmi1−/− -derived satellite cells do not efficiently re-enter the cell cycle when returned to high serum conditions after 5 days in low serum differentiation conditions (B). There are also fewer proliferating cells in the growth conditions and the cells quickly drop of division and differentiate after switching to low serum differentiation conditions (B). These data raise the possibility that Bmi1−/− satellite cells become senescent (B; n = 4). * <P0.05%, ** <P0.01% and *** <P0.001.

We have shown that Bmi1 is expressed in the satellite cells and that Bmi1 deficiency leads to reduced Pax7+/Myf5− stem cell pool. Yet we cannot exclude that the impaired regeneration observed is due to environmental effects rather than to a cell intrinsic defect. To begin to distinguish among these options, satellite cell cultures were derived from single fibers isolated from the SOL muscle of P60 Bmi1−/− and control littermates and induced to differentiate. Two days later, cells were labeled for Pax7, MyoD and MyHC and results quantified. We show that Bmi1−/− cultures contained very few Pax7+ cells and relatively more MyoD+ cells (Fig. 7A) mirroring the increased MyoD+ cells seen *in vivo*. Similar results were obtained also at P7 (Fig. 7A).

Next, we investigated whether the results obtained could be explained through Bmi1 modulation of satellite cells proliferation. Satellite cells obtained from P60 single fibers of the SOL were treated with BrdU in four different culturing conditions: growth (2 days in proliferation medium), early differentiation (2 days in differentiation medium), late differentiation (5 days in differentiation medium), and re-stimulation (2 days of proliferation medium after 5 day of differentiation, Fig. 7B).

Significantly fewer BrdU positive Bmi1−/− satellite cells were counted under high serum proliferative stimulation (14% BrdU positive in the Bmi1−/− compared to 43% BrdU positive in the wild type), while no difference in the percentage of BrdU positive cells was noted after 2 or 5 days of differentiation. Interestingly, a similar impairment in the proliferative capacity, as assessed by BrdU incorporation, was observed when Bmi1−/− satellite cells were returned to high serum conditions (proliferation medium) after 5 days in low serum conditions (differentiation medium) (Fig. 7B).

These results show that Bmi1 plays an important role in satellite cells proliferation and that its deficiency particularly affects the uncommitted satellite cell pool, which is significantly depleted, in keeping with a cell intrinsic effect of Bmi1 on satellite cell function.

## Discussion

Despite recent advances in elucidating cells and molecular pathways involved in muscle regeneration upon injury and in myopathic conditions, much remains to be understood before effective therapeutic intervention may be designed to sustain and enhance regeneration. In this study, we show for the first time that the PcG gene Bmi1 is expressed in human and murine satellite cells. Moreover, we demonstrate that Bmi1 plays a crucial role in controlling proliferation and self-renewal of these cells and its loss significantly impairs muscle homeostasis and regeneration upon injury.

Bmi1 deficiency had no effect on the patterning and differentiation of skeletal muscle during embryonic, fetal stages of myogenesis and early postnatally, in keeping with previous findings in other organ systems which have demonstrated a significant redundancy among PcG genes during embryonic development [38].

However, we show here a striking reduction in the number of Pax7+ satellite cells at P7, a loss that has a significant impact on the size of the muscle fibers. Consequentially, the postnatal growth of the skeletal muscle was severely affected, contributing significantly to the dramatic growth retardation observed in these mice [27]. Moreover, the remaining satellite cells displayed a reduced capacity to proliferate *in vitro* even under high serum conditions. Pax7+ satellite cells comprise two subpopulations: a stem cell population, which is Myf5− and a myogenic committed population, which is Myf5+. Interestingly, we found that while the number of Pax7+/Myf5− stem cells is depleted in Bmi1−/− muscle, the number of Pax7+/Myf5+ committed myogenic precursors and of MyoD+ activated myogenic precursors is increased. In normal postnatal muscle, most of the satellite cells are Pax7+ and very few cells are MyoD+ [11], [39], reflecting the normal low level of regenerative activity going on in adult muscles. However, when muscle is injured, the regenerative process is activated and MyoD is rapidly induced [8]. Similarly, satellite cells isolated from single myofibers and cultured under proliferative conditions, have been shown to rapidly upregulate MyoD [40], [41]. Upon switching to differentiation inducing culturing conditions, a small population of myogenic cells will down-regulate MyoD (retaining Pax7) and will remain undifferentiated and enter a mitotically inactive state, in keeping with the quiescent satellite cells *in situ* [42], [43]. The reduced number of Pax7+ cells together with the increased number of MyoD+ found in Bmi1−/− muscle both *in vivo* and *in vitro* suggests that the repopulation of the stem cell pool is impaired in Bmi1−/− animals. In keeping with this interpretation, we found that, although regeneration after a single injury is less efficient in Bmi1−/− mice, this becomes an even more pronounced regeneration defect after repeated injury, a situation that is dependent on the repopulation of the satellite cell pool.

The freeze injury model is a very well characterized model of traumatic muscle injury, indeed it affects not only the muscle fibers but also the supporting structures, such as the fibrocollagenous tissue and the resident histiocytic populations [32], [44], [45]. The Mdx mouse instead is a model of a muscle specific degenerative condition mimicking the human dystrophinopathy of Duchenne Type [35], [36], [37], [46], [47]. We show here that Bmi1 is essential for efficient regeneration of the skeletal muscle in both pathological conditions. Although we cannot exclude a contribution of cell populations other than the satellite cells to this phenotype, the defect in proliferation and the impaired ability to re-enter the cell cycle after differentiation, as well as the decreased ability of the Bmi1 deficient satellite cells to repopulate the stem cell pool observed in pure satellite cell cultures support the conclusion that satellite cells depend on Bmi1 for their biological and homeostatic functions.

Acute ablation of Bmi1 in myoblasts by means of miRNA followed by induction of myogenic differentiation led to severe reduction of the number of differentiated myotubes arising at the end of the differentiation time-course and to impairment of their alignment and fusion [48]. Although we do note that the differentiated Bmi1−/− myocytes *in vitro* tend to be shorter than the control myocytes differentiated for the same time (data not shown), we do not find impairment of myogenesis in our experimental setting either *in vitro* or *in vivo*. Currently, we do not have an explanation for this different outcome, it can be speculated that either the developmental time point at which Bmi1 deficiency is achieved or the specific subpopulation of cells targeted or gene dosage, may all contribute to explain these differences.

In conclusion, we show that Bmi1 regulates satellite cell functions both *in vitro* and *in vivo*. Satellite cells are the main stem cell reservoir of the adult skeletal muscle and they play a crucial role in regeneration of the muscle after injuries and during degenerative muscular conditions, such as muscular dystrophies and during the ageing process. Understanding the mechanisms regulating satellite cell function will therefore also be a key for developing novel therapeutic strategies for these conditions.

## Supporting Information

Figure S1 **No effect on the number of fibres and Pax7 positive cells in E16.5 Bmi1−/− embryos as compared to wildtype littermates.** There is no significant difference in the proportion of Pax7 positive cells found in the E16.5 Bmi1−/− embryos in the three regions counted, the lattisimus dorsi muscle (shoulder), the diaphragm and the calf muscles of the lower limb (limb).
(TIF)

Figure S2 **No changes in the expression levels of other PcG genes in Bmi1−/− satellite cell cultures.** qRT-PCR analysis of satellite cells isolated from Bmi1^−/−^ shows no significant alterations in the relative expression levels of other PcG genes known to play a role in myogenesis.
(TIF)
