# Supplementary figures and images for: Bmi1 Is Expressed in Postnatal Myogenic Satellite Cells, Controls Their Maintenance and Plays an Essential Role in Repeated Muscle Regeneration
Source: PLoS One. 2011 Nov 9;6(11):e27116. doi: 10.1371/journal.pone.0027116 (PMC3212532; doi:10.1371/journal.pone.0027116)

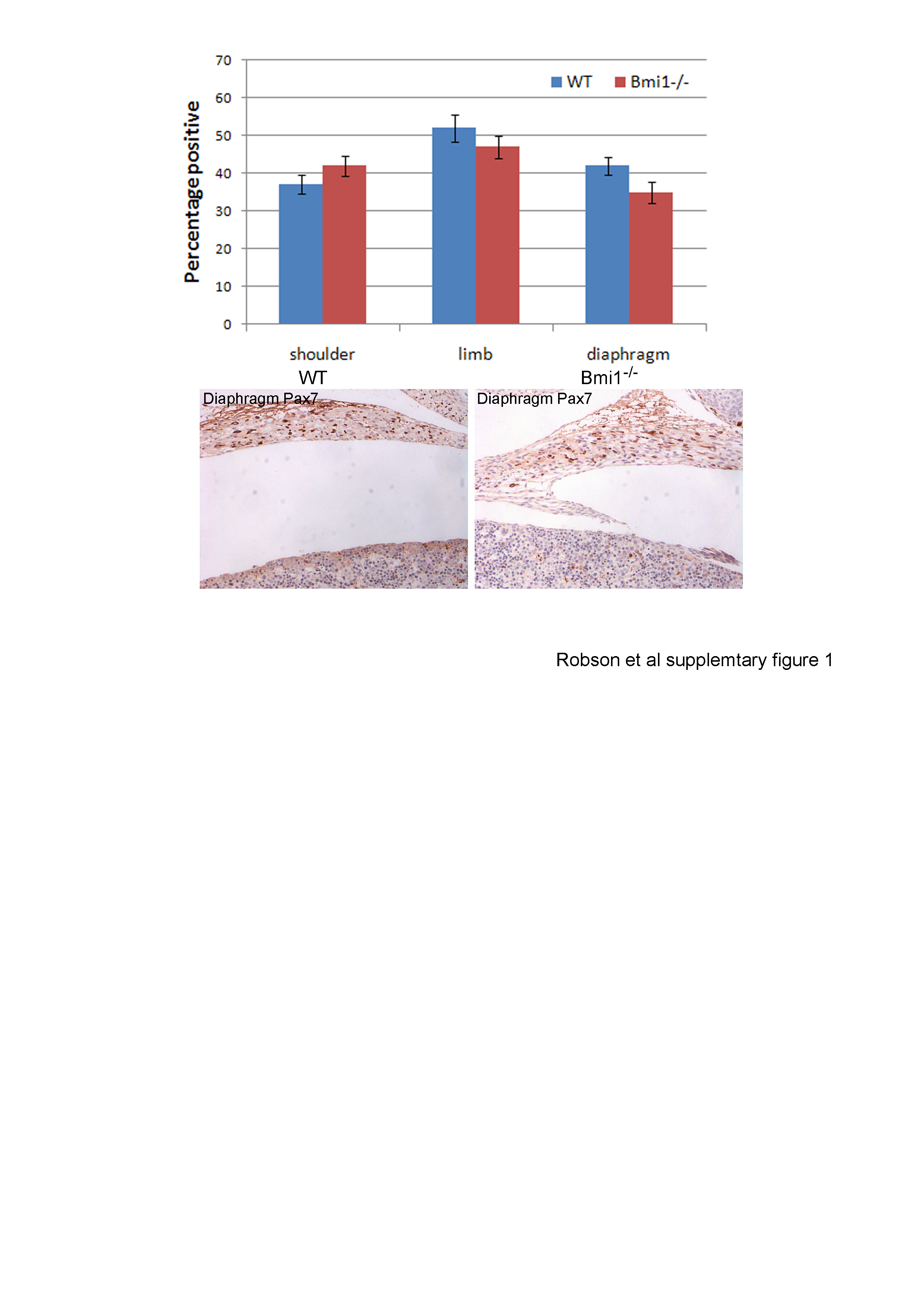

Supplement: Figure S1 — No effect on the number of fibres and Pax7 positive cells in E16.5 Bmi1−/− embryos as compared to wildtype littermates. There is no significant difference in the proportion of Pax7 positive cells found in the E16.5 Bmi1−/− embryos in the three regions counted, the lattisimus dorsi muscle (shoulder), the diaphragm and the calf muscles of the lower limb (limb). (TIF) [file pone.0027116.s001.tif]

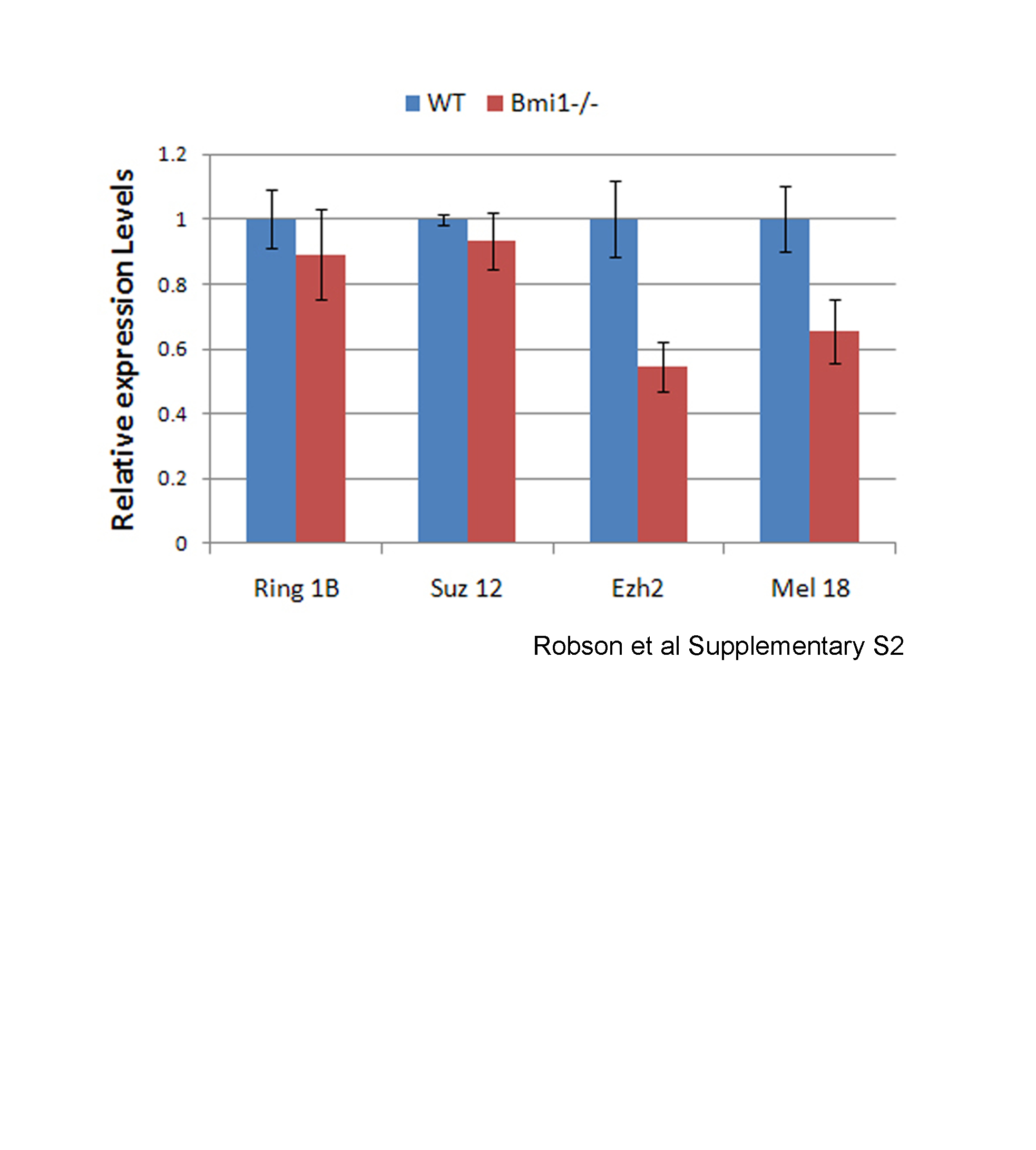

Supplement: Figure S2 — No changes in the expression levels of other PcG genes in Bmi1−/− satellite cell cultures. qRT-PCR analysis of satellite cells isolated from Bmi1−/− shows no significant alterations in the relative expression levels of other PcG genes known to play a role in myogenesis. (TIF) [file pone.0027116.s002.tif]
